# Mutations Utilize Dynamic Allostery to Confer Resistance in TEM-1 β-lactamase

**DOI:** 10.3390/ijms19123808

**Published:** 2018-11-29

**Authors:** Tushar Modi, S. Banu Ozkan

**Affiliations:** Department of Physics and Center for Biological Physics, Arizona State University, Tempe, AZ 85287-1504, USA; tmodi@asu.edu

**Keywords:** β-lactamase, allostery, evolution, antibiotic resistance, conformational dynamics, flexibility, dynamic coupling, molecular dynamics

## Abstract

β-lactamases are enzymes produced by bacteria to hydrolyze β-lactam antibiotics as a common mechanism of resistance. Evolution in such enzymes has been rendering a wide variety of antibiotics impotent, therefore posing a major threat. Clinical and in vitro studies of evolution in TEM-1 β-lactamase have revealed a large number of single point mutations that are responsible for driving resistance to antibiotics and/or inhibitors. The distal locations of these mutations from the active sites suggest that these allosterically modulate the antibiotic resistance. We investigated the effects of resistance driver mutations on the conformational dynamics of the enzyme to provide insights about the mechanism of their long-distance interactions. Through all-atom molecular dynamics (MD) simulations, we obtained the dynamic flexibility profiles of the variants and compared those with that of the wild type TEM-1. While the mutational sites in the variants did not have any direct van der Waals interactions with the active site position S70 and E166, we observed a change in the flexibility of these sites, which play a very critical role in hydrolysis. Such long distance dynamic interactions were further confirmed by dynamic coupling index (DCI) analysis as the sites involved in resistance driving mutations exhibited high dynamic coupling with the active sites. A more exhaustive dynamic analysis, using a selection pressure for ampicillin and cefotaxime resistance on all possible types of substitutions in the amino acid sequence of TEM-1, further demonstrated the observed mechanism. Mutational positions that play a crucial role for the emergence of resistance to new antibiotics exhibited high dynamic coupling with the active site irrespective of their locations. These dynamically coupled positions were neither particularly rigid nor particularly flexible, making them more evolvable positions. Nature utilizes these sites to modulate the dynamics of the catalytic sites instead of mutating the highly rigid positions around the catalytic site.

## 1. Introduction

Since the introduction of penicillin in the 1940s, β-lactam antibiotics are the most popular antibiotic agents, accounting for about 65% of all antibiotic consumption across the world [1,2,3,4]. β-lactams target enzymes that synthesize the bacterial cell wall. They constitute an effective low-cost method to treat infections. As a protective measure, TEM-1 β-lactamase enzymes synthesized in bacteria open the β-lactam ring through hydrolysis, inactivating the antibiotic. Therefore, β-lactamases constitute the major defense mechanism against β-lactam based antibiotics, particularly for Gram-negative bacteria [4]. They confer resistance to penicillin and early cephalosporins and have been shown to exhibit functional plasticity in response to the introduction of novel drugs derived from these antibiotics. Since its discovery in the early 1960s, over 170 variants of TEM-1 have been isolated in hospitals and clinics [5]. Their high evolvability has also made them one of the most studied enzymes through laboratory evolution experiments in an effort to understand the natural evolution of resistance as well as predict resistance driving mutations [2,5,6,7].

A comparison of the amino acid substitutions found in all known clinical TEM-1 isolates with those found in the in vitro or in vivo experimental evolution of TEM-1 revealed that the substitutions that were found with a high frequency in clinical isolates overlapped with the resistance driving mutations of the laboratory experiments (i.e., E104K, R164C, R164H, R164S, A237T, G238S, and E240K). However, there are a significant number of mutations that have been observed in the laboratory evolution experiments, but have not been found in clinical isolates. Moreover, the structure alone did not help explain the molecular mechanism by which these mutations contribute to antibiotic resistance. Indeed, the mutational landscape that drives β-lactamase enzymes toward more specific functions is extremely complex and multi-dimensional [8,9,10,11] and incorporates a large number of neutral, deleterious, and beneficial mutations distributed throughout the sequence space of the enzymes. While several of these mutations are close to the active site in the 3D structure, having direct interactions with the active site, many of the mutations that contribute to resistance are distally located. A number of studies have been performed on clinical and laboratory isolates of mutations that showed that mutations allosterically modulated the degradation of antibiotics and binding of inhibitors [12,13,14,15,16,17].

Despite the success in showing the allosteric impact of resistant mutations [14,15], the general mechanism of the allosteric modulation of activity by these mutations is still unknown. In particular, predicting the mutational sites beforehand that could be observed in clinical isolates for resistance is still very challenging. It is essential to understand the highly complex relationship between mutations and the active sites in β-lactamase enzymes to design more efficient antibiotic therapeutics. To answer the question of whether there are generalized molecular mechanisms for the positions that contribute to resistance, we explored the role of conformational dynamics. The analysis of laboratory-resurrected ancestral β-lactamases revealed that they were also resistant to antibiotics, and in fact could degrade not only the first-generation antibiotics (e.g., penicillin), but also the later generation ones with the same efficiency [2,6]. Moreover, a comparison of the conformational dynamics of ancestral β-lactamases with penicillin-specific extant TEM-1 has shown that the ancestral proteins have an increased conformational diversity to degrade a variety of antibiotics [2]. Indeed, in all of our resurrected ancestral protein studies, we observed that nature fine-tuned the native state ensemble to evolve to a new function or to adapt to a new environment [2,18]. Here, we also aimed to compare the conformational dynamics and the role of dynamic allostery in resistance driving mutations that have been frequently observed in both the clinical isolates and laboratory evolution experiments [5]. Furthermore, we investigated whether the similar principle of “fine-tuning of conformational dynamics” dictates the positions that are substituted for resistance. To investigate this, we applied the Dynamic Coupling Index (DCI) and Dynamic Flexibility Index analysis (DFI) to TEM-1. DFI is a metric that qualitatively measures the relative conformational entropy (flexibility) of a residue position with respect to the rest of the protein chain. On the other hand, DCI gives a quantitative score of the long range dynamic communication between a given position and the active site. We are particularly interested in the DCI profiles of the positions that are observed as being frequently mutated in the clinical isolates.

We observed that a majority of single point mutations found in clinical isolates and laboratory evolution experiments [5] have a higher coupling with the active sites (a high DCI score). In addition, many of these sites were found to impact the function allosterically by modulating the flexibility of the active site. Moreover, in order to test the completeness of the approach, we also examined the data from an exhaustive study [19] that provides the fitness landscape of TEM-1 β-lactamase with respect to the surviving populations of bacteria under a selection pressure of different concentrations of the antibiotics ampicillin and cefotaxime upon performing all possible single point substitutions. By applying our dynamics-based metrics on this dataset, we observed a similar trend where the mutations with a higher impact on the fitness also exhibited a higher DCI score with the active site, indicating that positions contributing to resistance indeed have a long range dynamic coupling with the active sites. In addition, these positions also belong to regions with mid-range to higher flexibility in the protein that allow them to be more robust to mutations. Additionally, deleterious mutations are typically located at positions with low flexibility, and high flexibility regions house neutral mutations [20,21,22,23]. Overall, we observed a generic behavior in evolution [18] of TEM-1 where mutations were more frequently observed in regions exhibiting medium flexibility and high dynamic coupling with the active site to fine-tune the dynamics of the active site modulating the function.

## 2. Results and Discussion

We analyzed the impact of resistance driving single point mutations that have been most frequently observed in a large number of both clinical and laboratory isolates of evolution experiments of TEM-1 β-lactamase, compiled by Salverda et al. (2010) (Table 2 in [5]). These mutations modulate the binding of inhibitor and/or β-lactam antibiotics and give rise to antibiotic resistance [24]. While they alter the β-lactamase function to degrade different antibiotics, the experimental biophysical characterization of these antibiotic resistant variants has shown that the mutations do not change the 3D structure of the protein and also yield similar protein expression levels [2,6]. This leads to the question of how these mutations can alter the function of the protein while conserving their 3D fold.

### 2.1. A Majority of the Resistance Driving Mutations Are Distal to the Active Site

**Figure 1:**
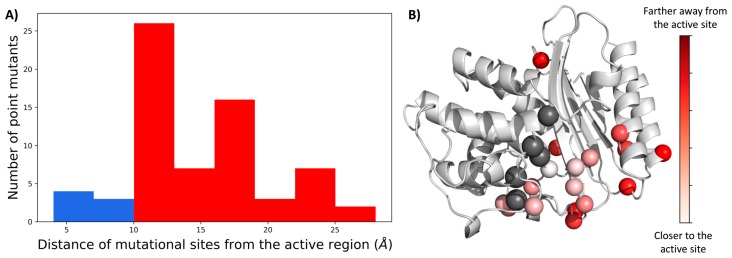
(**A**) Distribution of the distance of mutational sites in single point mutations observed in clinical isolates and laboratory evolution experiments in wild type TEM-1 β-lactamase around the active site. It was observed that the majority of the mutations were distal to the active site (i.e., >10 Å shown in red) and hence impacted the function via allosteric interactions. On the other hand, a very small number of variants were observed where mutational sites were closer to the active site (in blue) (**B**) Cartoon representation of TEM-1 with active sites in black spheres and the mutational sites studied in colored spheres, where the color depends on their distance from the centroid of the catalytic region.

First, we mapped the locations of all mutational substitutions observed in clinical and laboratory isolates of TEM-1 [5] on the 3-dimensional structure of TEM-1 and calculated the radial distribution of the location of each mutational position from the center of the catalytic region (S70, S130, N132, E166, and K234). Interestingly, we observed that the majority of these mutations lay farther than 10 Å away from the catalytic site (Figure 1), which is out of range for direct contact with the active sites. Therefore, it indicates that they must alter the function by allosterically modulating the network of interactions in the protein chain. This suggested mechanism of antibiotic resistance driven by single point mutations is in agreement with our previous work on the functional evolution of GFP proteins, Thioredoxins, β-lactamases, etc. [2,18,20,25,26], showing that mutations critical for the emergence of a new function are usually farther away from the active site, and manipulate the function by fine-tuning the native state ensemble while conserving the 3D fold in the native state. Through other studies, it is now well established that mutations endowing an enzyme with a novel function are generally destabilizing [27]. Therefore, it is suggested that enzyme adaptation to a novel function should require both functionally beneficial but thermodynamically destabilizing mutations, and compensatory stabilizing mutations. The complex epistatic relationship among these mutations allows for the emergence of a novel function. Several studies involving in vitro laboratory evolution [28,29] and computational techniques employing the use of Markov state models [12,13], statistical models [7,8,9,30], structure [31], and dynamics-based models [18] have been performed where the epistatic effects of pairs of mutations in evolutionary pathways have been explored and the role of allosteric mutations has been emphasized including β-lactamases [12,13,14,15,16] and other enzymes [32,33,34,35]. In particular, it has been shown that the thermodynamic effects of beneficial mutations are uncorrelated with cefotaxime resistance in TEM-1 β-lactamase [10]. Through the analysis of conformational dynamics, we further explored the molecular mechanism by which these single point mutations modulate the resistance while keeping the 3D fold conserved.

### 2.2. Antibiotic Resistance Driving Single Point Mutations Alter the Flexibility Profile of TEM-1 β-lactamase

We divided the dataset into two parts: (i) the mutations that drive inhibitor resistance, N276D, R244C, R244S, R275L, R275Q, and S268G; and (ii) the mutations that drive the resistance of β-lactam antibiotics, E240K, I173V, Q39K, and S268G. We analyzed the flexibility profiles of the wild type and the resistant variants of TEM-1 using the Dynamic Flexibility Index, DFI [2,21,26] (see Methods). DFI is a position specific metric which gives the measure of relative resilience of an amino acid to random force perturbations, mimicking the natural condition of a protein in a crowded cell. Thus, it is related to the contribution of each position to the conformational entropy of the protein, and also quantifies the relative flexibility of each amino acid position with respect to the rest of the residues in the protein.

**Figure 2:**
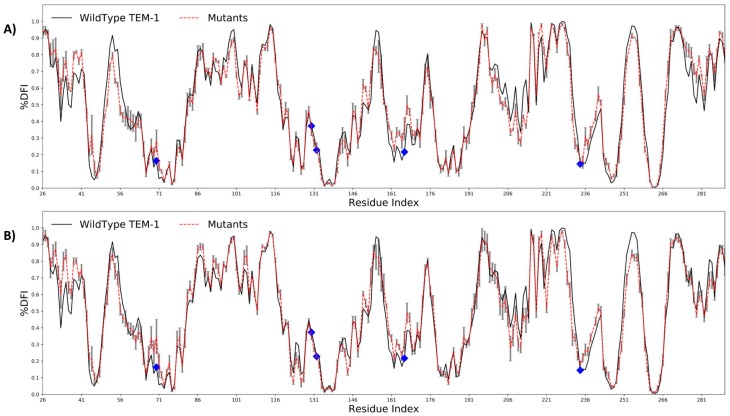
The difference in the conformational dynamics of the wildtype TEM-1 β-lactamase (black) with the average %DFI profile (y-axis) of the single point variants (red), distal from the catalytic region (blue diamonds). We found that the mutants, despite being distal to the active region (Figure 1) impacted the dynamics of the active sites, particularly S70 and E166. This behavior was observed in the single point variants providing resistance to β-lactam antibiotics (**A**) as well as in the mutants impacting the resistance through inhibitors (**B**).

The DFI profiles of the wildtype TEM-1 and its mutants were obtained using all atom MD simulations (see Section 3). We compared the DFI profile of the wild type with the averaged DFI profile of two sets of mutants (Figure 2A,B). Both types of resistance driving mutations exhibited the same behavior. They altered the flexibility of hinge regions (i.e., rigid sites), particularly the catalytic sites S70 and E166, despite that fact that the mutations did not have any direct interaction with the active sites.

Positions exhibiting low DFI values are usually those that play a critical role in transferring the perturbations throughout the protein. Similar to hinges in a door frame or joints in a skeleton, these positions are necessary for mediating collective motion dynamics, which are important for the protein to function. Our previous studies on the evolution of Thioredoxin, GFP, etc. [2,18,20] have shown that (i) the mutations that alter the flexibility of these hinge regions (i.e., low DFI sites) lead to changes in the protein function, and (ii) nature also fine-tunes the flexibility of these hinge regions through mutations leading to the emergence of new function. Similarly, other studies have also shown that mutations altering the conformational dynamics play a key role in the evolution of a new function [36]. In alignment with the above-mentioned studies, it was interesting to see a change in the DFI values of the hinge site S70. This site plays a critical role in the catalytic mechanism by serving as the nucleophile for attack on the carbonyl carbon of the amide bond. The change in DFI profile of S70 suggested that these allosteric mutations fine-tune the flexibility of the catalytic positions by altering their conformational dynamics for the hydrolysis of new antibiotics. Likewise, the same mechanism (i.e., the change in the flexibility of active sites that play a major role in binding and catalysis) was also observed for the second set of mutations, which are known as mutation driving the resistance through inhibitors (Figure 2B, Supplementary Figures S1 and S2).

**Figure 3:**
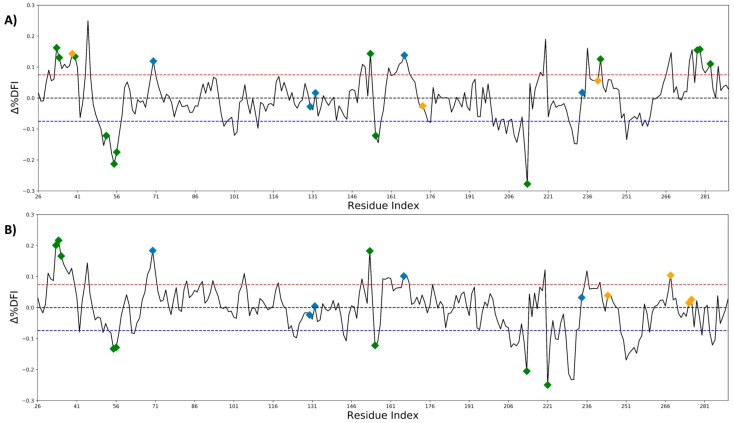
Comparison of the conformational dynamics of the wild type TEM-1 β-lactamase with the average DFI profile of the single point variants, distal from the catalytic region (blue diamonds), by calculating the difference in their %DFI profiles (Δ%DFI, y-axis) of the mutants impacting the hydrolysis of β-lactam antibiotics (**A**) and the mutations driving resistance through inhibitors (**B**). The red and blue dash line represents ±1.0 times the standard deviation around mean (black dash line) of the differences between the profiles. We observed that the mutations (orange diamonds) impacted the dynamics of the catalytic region particularly around S70 and E166. In addition, we observed that many of the single point mutations explored in laboratory evolution experiments were found in regions with significant differences in their conformational dynamics when compared to the wildtype (green diamonds) [5]. This hints towards their possible contribution to the dynamics of the catalytic region.

These observations were consolidated by calculating the change in the flexibility profile of TEM-1 post mutations. We obtained the change in DFI profile (∆DFI) by comparing the averaged DFI profiles of the mutants with that of the wild type. In particular, we focused on the positions that exhibited a significant change in DFI, i.e., more than one standard deviation of change than the average change in ∆DFI (Figure 3A,B). Interestingly, this analysis revealed that while the DFI profile of most of the actual mutational sites studied in the present work did not change significantly (orange diamonds), it triggered changes in the DFI values of the distal positions, particularly near the active site (blue diamonds, of note S70 and E166). Furthermore, we also discovered that some of the positions that exhibited significant change upon resistance driving mutations from clinical isolates corresponded to the mutational points that had already been observed in the laboratory evolution of TEM-1 (Figure 3A,B green diamonds). However, further analysis is required to understand the underlying mechanism governing antibiotic resistance emerging from these mutations.

### 2.3. Dynamic Coupling Index (DCI) Gives an Insight into the Internal Network of Interactions in TEM-1 β-lactamase Protein

Since we observed that our analyzed mutations providing antibiotic resistance to TEM-1 β-lactamase allosterically modulated the flexibility of the active sites, we measured their long-distance dynamic interactions with the active sites using a metric called the Dynamic Coupling Index (DCI) [37,38]. DCI utilizes linear response theory and perturbation response scanning to measure the strength of the coupling interaction between any pair of residues or between a residue and a region (i.e., active sites) (see Section 3). It helps identify the dynamic allosteric residue coupling (DARC) spots [38], which are sites that are distal to active sites but remotely regulate them through modulating the flexibility of functionally critical sites. This type of allosteric coupling, regardless of the distance of separation, is also likely to contribute to the function.

**Figure 4:**
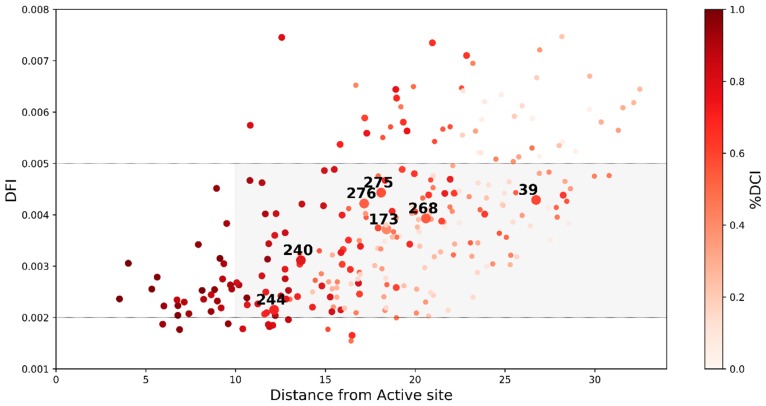
A distribution of flexibility of various residue locations in TEM-1 β-lactamase given by their DFI score (y-axis) with their distance from the catalytic region (x-axis). The residues are colored in accordance to the strength of their coupling with the active sites using their %DCI score with red being most coupled (%DCI = 1) to white without any coupling (%DCI = 0). We observed that a large number of the mutations analyzed in clinical isolates and laboratory evolution experiments of TEM-1 had a strong coupling (represented by bigger circles) regardless of being distal to the active sites. Moreover, these mutations, along with many other residue positions coupled to the active site, lay in a region of medium flexibility in the protein.

We observed that, irrespective of the separation distance between the mutational sites and the active sites (Figure 1 and Figure 3), most of the resistance driving mutations we studied were dynamically coupled with the active sites as shown by their overall higher DCI scores (Figure 4, color-coded where darker spots exhibit high DCI values). These DARC spots exhibited mid-range flexibility (i.e., inside the gray shaded area). The increased flexibility of these sites makes them more robust to the impact of mutations, while the high dynamic coupling of these residues with the actives site enables them to remotely modulate active site flexibility. This observed mechanistic picture is in agreement with our previous analysis of missense variants, the disease associated mutations on the DARC spots regulate the flexibility and dynamics of functionally critical sites from a distance leading to a loss or gain in the protein function [37,38,39].

**Figure 5:**
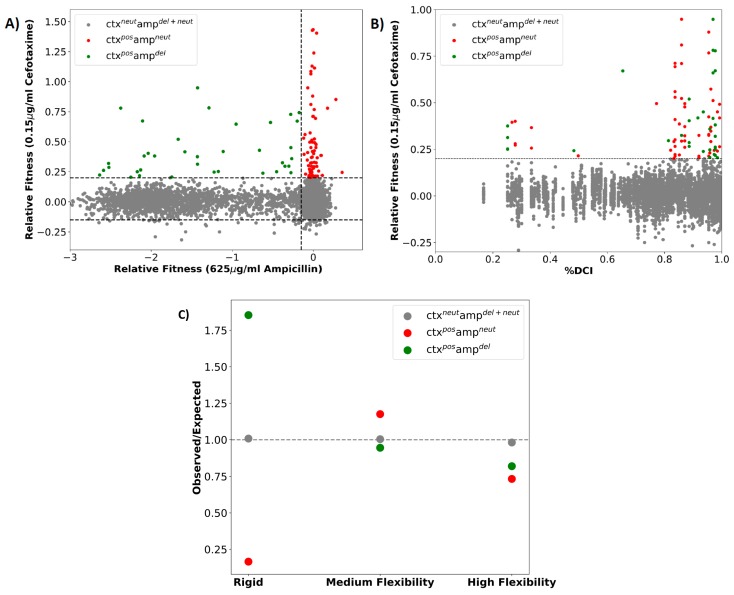
(**A**) Relative fitness values for all mutations under ampicillin (625 mg/mL (x-axis) versus CTX selection (0.15 mg/mL; y-axis) showed that most mutations conferring significant CTX resistance were mostly neutral (red), but some were deleterious (green) in ampicillin resistance, while the color grey shows the variants with no effect on cefotaxime resistance. (**B**) Change in the relative fitness of the organism upon various mutations as a function of their %DCI couplings with the active site. We observed that a majority of the mutations with positive fitness in CTX were highly coupled to the active region (%DCI > 0.7). (**C**) Observed to expected ratio of TEM1 variants based on their flexibility category.

In observing the DCI scores of all of the residue locations in TEM-1 (Figure 4) we noticed that, in addition to the mutational sites found in clinical and laboratory isolates, there were a large number of other residue locations in TEM-1 with a high DCI coupling score with the active site. Many of these residue locations were distal from the active sites and in a region of medium flexibility (gray region in Figure 4). According to our analysis of other proteins, mutations at these residue locations may serve as allosteric modulators for the function without compromising the fold or stability while making subtle changes in the dynamics of active sites [18,20]. Therefore, there is a need to perform an exhaustive mutagenesis scan of all possible mutations at each residue location in TEM-1 in order to analyze the impact of mutations at these sites on the activity of the protein. Similar in vivo experiments were performed by Stiffler et al. 2015 [19] where they investigated the impact of all possible single point mutations (4997 mutations) in TEM-1 on the organism’s fitness under the selection pressure for ampicillin resistance (i.e., the wildtype function) and cefotaxime (CTX) resistance (i.e., evolving a new function) (Figure 5A). Upon mapping the fitness data over the DCI profile of the protein, we observed that a significant number of residues with mutations with a positive effect on the fitness in the CTX antibiotic were highly coupled to the active sites (%DCI > 0.7) (Figure 5B). This observation is in agreement with the analysis shown above where the mutations that drive resistance to an antibiotic should exhibit high dynamic coupling with the catalytic sites, irrespective of their distal location with the active sites.

Under the null hypothesis of no effect, the ratio of the observed to expected numbers of residue positions hosting variants increasing CTX fitness should be close to 1.0. While the null hypothesis was observed to be true for the ampicillin and CTX neutral mutations, it was completely rejected for the CTX positive variants. We observed that CTX-positive ampicillin deleterious variants were over abundant at rigid sites (ratio = 1.86), which is in agreement with our earlier studies where mutations at rigid sites were more deleterious when compared to wild type for the function (in this case ampicillin resistance). Moreover, in consensus with our earlier human proteome wide study, we observed that CTX-positive and ampicillin-neutral variants were over abundant at positions exhibiting medium flexibility (ratio = 1.21). Medium flexibility at these sites usually provides conformational freedom to accommodate the change in their interaction upon amino acid substitution. Furthermore, they also have more dynamic coupling with the active site when compared to sites with high flexibility. Therefore, the mutations at these sites can usually be neutral for ampicillin degradation, but help to evolve the emergence of a new function, CTX degradation.

Overall, the emergence of a new function (i.e., degradation of CTX) presented the same molecular mechanism that we observed in ancestral protein studies, i.e., nature modulates the flexibility of positions for the emergence of a new function [18,20,21,22,25,26,39]. In addition, our previous proteome-wide analysis showed that the evolutionary rate of positions was highly correlated with their flexibility [21]. A more detailed analysis on the evolution of Thioredoxins [18] indicated that evolution utilizes the residues belonging to mid-flexibility regions in proteins (0.002 ≤ DFI < 0.005) to introduce mutations and adapt to the new environment to function at higher pH and cooler temperatures. Therefore, our analysis on such protein systems suggests that evolution fine-tunes the native state ensemble as shown by the change in the distribution of the flexibility profile of the positions while keeping the fold conserved. We observed that the population of low flexibility rigid region (DFI < 0.002) and high flexibility regions (0.005 ≤ DFI) increased with time, and that of the medium flexibility region decreased. The same mechanism was found to be operational for the mutations driving CTX resistance in this large dataset, as shown by the observed to expected ratios (Figure 5C) of the population of low, mid, and high flexibility residues in TEM-1 β-lactamase.

## 3. Methods

### 3.1. Dynamic Flexibility Index

The Dynamic Flexibility Index (DFI) is a novel metric that quantifies the relative local vibrational entropy of residues in a protein structure. It utilizes a perturbative force as a probe, which scans over a protein chain through Perturbation Response Scanning (PRS) [22,25]. Here, the protein is modeled as an elastic network with residues represented by a node at their alpha-carbon atom and the interacting residues are connected by harmonic springs. Using linear response theory [25,40,41,42,43,44] and a Brownian kick as a perturbative force, the displacements of the nodes can be calculated as:

(1) $$\Delta R_{3N\times 1}=H_{3N\times 3N}^{-1}F_{3N\times 1}$$

where **H**^−1^ is the Hessian inverse **3*N*** × **3*N*** matrix of the network composed of the second order derivatives of the harmonic potential with respect to the components of the position vectors for the chain of length *N*, giving the position co-variance of the residue pairs in equilibrium; **F** is the perturbative force vector; and **Δ*R*** is the response vector of residues due to force. However, even though ENM based PRS models can very accurately capture the response fluctuations arising from low frequency modes, these are not sensitive to the biochemical specificity of amino acids. Therefore, ENM based models fail to capture the differences in dynamics between mutants with similar native state structure. Hence, to incorporate the biochemical specificity, the Hessian inverse can be replaced with a covariance matrix, ***G***, obtained from all-atom MD simulations [45] as:

(2) $$G_{3N\times 3N}=\frac{k_{B}T}{\gamma }H_{3N\times 3N}^{-1}$$

where *γ* is the force constant for the protein network. However, the multiplicative factors $\frac{k_{B}T}{\gamma }$ are constants for a given force vector and a protein network and were ignored, as in the end, we were interested in a metric that measured relative responses, and these common constants merely cancel out. Next, we calculated the response vector due to a perturbative force in the protein network as:

(3) $$\Delta R_{3N\times 1}=G_{3N\times 3N}F_{3N\times 1}$$

The covariance matrix incorporates the data for long range interactions, solvation effects, and biochemical specificities of all types of amino acids. Therefore, using the covariance matrix obtained from the molecular dynamics simulations, the difference in the dynamics of mutants with a similar 3-dimensional structure could be observed. Using the covariance matrix, the response profile of the protein can be calculated upon the application of random Brownian kicks in order to obtain an isotropic response. The displacements of the network calculated as random Brownian kicks were applied sequentially to each Cα atom in the elastic network in order to calculate the perturbation response matrix, ***A***, as

(4) $$A_{N\times N}=\left[\begin{array}{ccc} {\left|\Delta R^{1}\right|}_{1} & \cdots & {\left|\Delta R^{N}\right|}_{1}\\ \vdots & \ddots & \vdots \\ {\left|\Delta R^{1}\right|}_{N} & \cdots & {\left|\Delta R^{N}\right|}_{N} \end{array}\right]$$

where ${\left|\Delta R^{j}\right|}_{i}=\sqrt{\langle {\left(\Delta R\right)}^{2}\rangle }$ is the magnitude of the fluctuation response at site ‘*i*’ due to the perturbations at site ‘*j*’. A sum of a given row of perturbation matrix gives the net average displacement of the residue from its equilibrium position when all residues are perturbed by an isotropic unit force one at a time. The DFI score of a residue position ‘*i*’ is defined as the ratio of the net response of that position with the net displacement of the whole protein chain as all the residues are perturbed sequentially, i.e.,

(5) $$DFI_{i}=\frac{\sum_{j=1}^{N}{\left|\Delta R^{j}\right|}_{i}}{\sum_{i=1}^{N}\sum_{j=1}^{N}{\left|\Delta R^{j}\right|}_{i}}$$

Therefore, a higher DFI score implies that the site has a larger response when compared to the rest of the chain upon random perturbations, hence it is labelled as flexible. On the other hand, a lower DFI score means that the residue location has a smaller response when compared to the rest of the protein chain to random perturbations, and is hence labelled as rigid.

### 3.2. Dynamic Coupling Index

The Dynamic Coupling Index (DCI) [23,38,39] is a metric that quantifies the coupling interaction between two residues in a protein. Like DFI, it is also based on principles like PRS and linear response theory. DCI for a residue ‘*i*’ with another residue ‘*j*’ is defined as the ratio of total displacement at residue ‘*i*’ when Cα at residue ‘*j*’ is perturbed by a unit Brownian kick, to the average displacement of residue ‘*i*’ when all the residues in the protein chain are perturbed by unit force Brownian kicks. Therefore, it can be used to measure the coupling of a residue with functionally important sites as:

(6) $$DCI_{i}=\frac{\sum_{j}^{N_{functional}}{\left|\Delta R^{j}\right|}_{i}/N_{functional}}{\sum_{j=1}^{N}{\left|\Delta R^{j}\right|}_{i}/N}$$

where |**∆*R****^j^*|*_i_* is the response fluctuation profile of residue ‘*i*’ upon the perturbation of residue ‘*j*’. The numerator is the average mean square fluctuation response obtained over the perturbation of the functionally critical residues *N_functional_* and the denominator is the average mean square fluctuation response over all residues. Here, we can also use the covariance matrix obtained from MD simulations to incorporate the dynamics from all atomic interaction potentials.

As a result, a higher DCI score of a residue implies that perturbation on functionally important sites have a higher response at the residue location in comparison to the rest of the protein and is thus labelled as more coupled, whereas a low DCI score would mean the opposite.

### 3.3. Molecular Dynamic Simulations

In order to analyze the impact of single point mutations, we performed all atom MD simulations of the wild type and its variants. The starting structure of the wild type TEM-1 was obtained from the protein databank (PDB id: 1BTL [46]). Subsequent variants were created using the mutagenesis tool of PyMol [47] by replacing the wild type amino acid with the mutant amino acid template where the initial rotameric state is selected in order to have minimum steric hinderance. The structures were then loaded into TLEAP (Amber) to obtain the topology using the forcefield ff14SB [48]. The protein hydrogens were added, and the protein was solvated in a 14 Å cubic water box (TIP3P) with neutralizing ions [49,50]. The system was then energy minimized using the SANDER module of Amber17 [51] by first minimizing the solvent molecules, followed by the entire solution. Heat-up, density equilibration, and production were all performed by the GPU accelerated PMEMD module of AMBER17 [51]. During these simulations, periodic boundary conditions were used, and the bond length of all covalent hydrogen bonds were constrained using SHAKE [52]. Direct-sum, non-bonded interactions were cut off at 9.0 Å, and long-range electrostatics were calculated using the particle mesh Ewald method [53,54]. The systems were heated from 0 to 300 K over a duration of 250 ps. Langevin and Berendsen thermostats were used for density equilibration and production at constant temperature and pressure of 300 K and 1 bar, respectively. A time step of 2 fs for the integrator was used for the heat-up and production run. A total of 600 ns of equilibrium production trajectories were obtained for the wild type and mutant proteins where output frames were saved every 10 ps. In order to obtain consistent DFI profiles of convergent simulations, the average DFI was computed using a running time average of different window sizes (25 ns, 50 ns, 75 ns, or 100 ns) such that the statistical agreement of hinges and flexible regions between the DFI profiles obtained from different window sizes was observed.

## 4. Conclusions

In this study, we investigated the impact of resistance driving single point mutations in TEM-1 β-lactamase. These mutations were observed in multiple clinical isolates of TEM-1 and in vitro laboratory evolution experiments compiled in Salverda et al. 2010 [5]. The mutations were responsible for providing antibiotic resistance to TEM-1 by either altering their activity with β-lactams or with inhibitors like β-lactam–inhibitor combination ampicillin–clavulanate [55]. Mapping their locations onto a 3D structure revealed that a large number of these mutations were distal to the active site (Figure 1) and hence might alter the activity of enzymes remotely via allosteric regulation.

Moreover, analyzing their Dynamic Flexibility Index (DFI) profiles revealed that the mutations altered the flexibility of the rigid parts of the protein, particularly around the active site S70, which plays a key role in the activity of the protein (Figure 2 and Figure 3). While the flexibility of the positions near the mutational site did not change significantly, we observed that these mutations remotely modulated the network of interactions near the active sites. This fine-tuning of the active site dynamics in β-lactamase might be responsible for the degradation of a novel substrate. This remote modulation between the allosteric mutational site and the active sites might be actualized via long distance dynamic coupling since a majority of the resistance driving mutation sites exhibited high dynamic coupling (DCI) with the active sites (Figure 4). These findings were further strengthened through an analysis of the dynamic coupling and flexibility of 4997 single point mutations that were characterized for the fitness landscape of CTX and ampicillin resistance. Upon mapping the DCI values of the mutational sites onto their fitness scores, we observed that, in agreement with our previous analysis, the mutations that altered the activity to a new function (activity with CTX in this case) were coupled to the active sites (Figure 5B). In addition, upon analyzing the flexibility of the mutating amino acid positions, we found that mutations belonging to the medium flexibility region led to the evolution of a new function (as seen from the observed to expected ratios, Figure 5C) as opposed to mutations in low flexibility regions where mutations were generally deleterious for ampicillin resistance. This, which is in complete agreement with our earlier investigation on the mechanism of evolution of Thioredoxin [18], points towards a central fact: evolution may utilize residues with medium flexibility to perform substitutions in order to fine-tune the flexibility of lowly and highly flexible sites in order to evolve a new function. In the process of doing so, it can exploit allosteric interactions in the network to change flexibilities remotely and compensate for the damaging effects of deleterious mutations by performing other mutations [18,56].

## Supplementary Materials

Supplementary Materials are available online at http://www.mdpi.com/1422-0067/19/12/3808/s1.
