# Supplementary material for: Mutations Utilize Dynamic Allostery to Confer Resistance in TEM-1 β-lactamase
Source: Int J Mol Sci. 2018 Nov 29;19(12):3808. doi: 10.3390/ijms19123808 (PMC6321620; doi:10.3390/ijms19123808)
Supplement: Supplementary file 1 [file ijms-19-03808-s001.pdf]

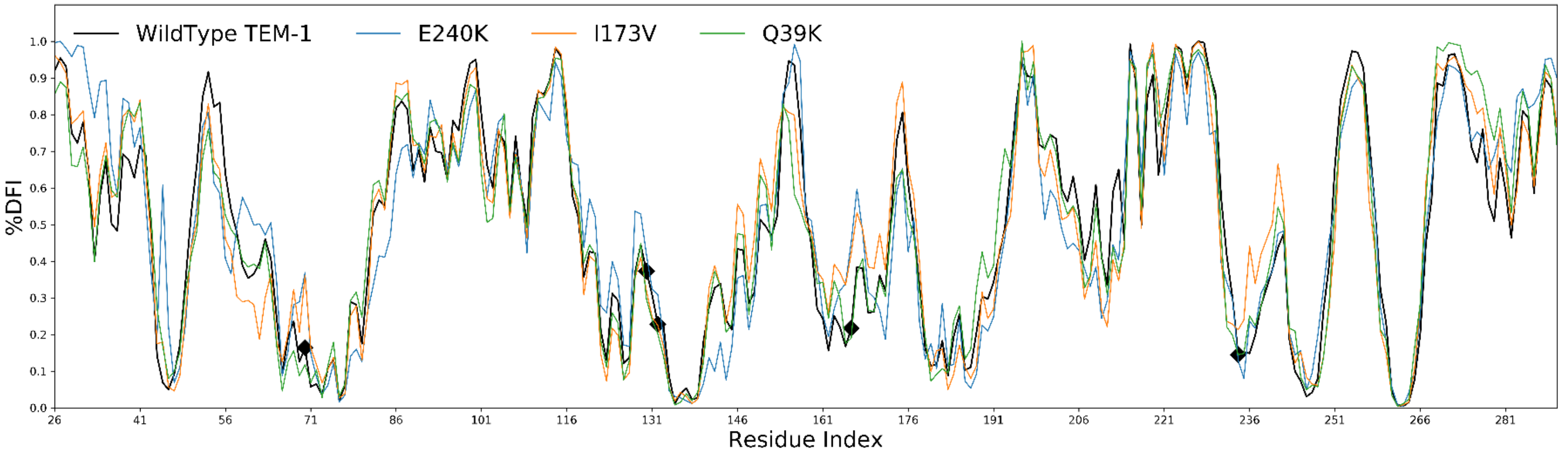


**Supplementary Figure 1.** Comparison of %DFI profiles of wildtype TEM-1 β-lactamase (Black) with the %DFI profiles of single point variants which provide resistance to β-lactam antibiotics. We observe that a majority of mutants, despite being distal to the active site region, alter the flexibility of the active site regions (in Black Diamonds) particularly at S70 and E166.

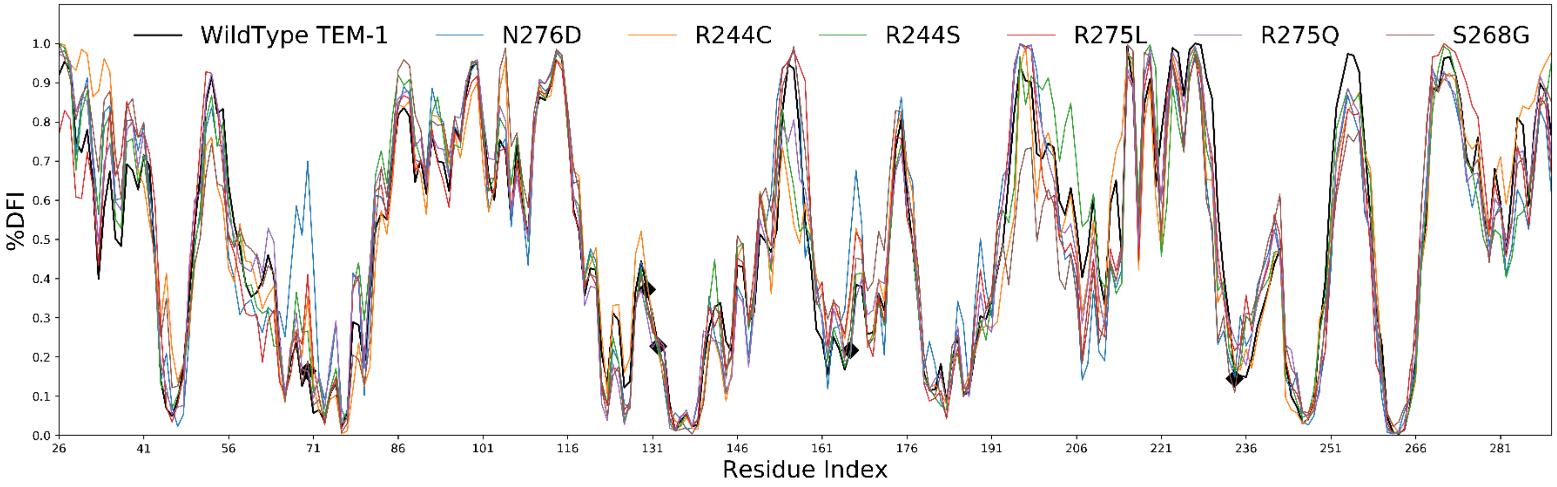


**Supplementary Figure 2.** Comparison of %DFI profiles of wildtype TEM-1 β-lactamase (Black) with the %DFI profiles of single point variants which impact antibiotic resistance through inhibitors. We observe that a majority of mutants, despite being distal to the active site region, alter the flexibility of the active site regions (in Black Diamonds) particularly at S70 and E166.
